# Can Genetic Testing Identify Talent for Sport?

**DOI:** 10.3390/genes10120972

**Published:** 2019-11-26

**Authors:** Craig Pickering, John Kiely, Jozo Grgic, Alejandro Lucia, Juan Del Coso

**Affiliations:** 1 Institute of Coaching and Performance, School of Sport and Wellbeing, University of Central Lancashire, Preston PR1 2HE, UK; craigpickering1014@hotmail.com (C.P.); jkiely@uclan.ac.uk (J.K.); 2 Institute for Health and Sport (IHES), Victoria University, Melbourne 3011, Australia; jozo.grgic@live.vu.edu.au; 3 Faculty of Sport Sciences, Universidad Europea de Madrid, 28670 Villaviciosa de Odón, Spain; alejandro.lucia@universidadeuropea.es; 4 Research Institute i+12, and Centro de Investigación Biomédica en Red de Fragilidad y Envejecimiento Saludable, 28041 Madrid, Spain; 5 Centre for Sport Studies, Rey Juan Carlos University, 28943 Fuenlabrada, Spain

**Keywords:** genetics, single nucleotide polymorphism, athletic performance, elite athlete, success

## Abstract

Elite athlete status is a partially heritable trait, as are many of the underpinning physiological, anthropometrical, and psychological traits that contribute to elite performance. In recent years, our understanding of the specific genetic variants that contribute to these traits has grown, such that there is considerable interest in attempting to utilise genetic information as a tool to predict future elite athlete status. In this review, we explore the extent of the genetic influence on the making of a sporting champion and we describe issues which, at present, hamper the utility of genetic testing in identifying future elite performers. We build on this by exploring what further knowledge is required to enhance this process, including a reflection on the potential learnings from the use of genetics as a disease prediction tool. Finally, we discuss ways in which genetic information may hold utility within elite sport in the future, including guiding nutritional and training recommendations, and assisting in the prevention of injury. Whilst genetic testing has the potential to assist in the identification of future talented performers, genetic tests should be combined with other tools to obtain an accurate identification of those athletes predisposed to succeed in sport. The use of total genotype scores, composed of a high number of performance-enhancing polymorphisms, will likely be one of the best strategies in the utilisation of genetic information to identify talent in sport.

## 1. Introduction

It is widely recognised that some individuals are naturally talented in specific physical traits that are, in turn, related to sports performance [1]. In some cases, talented individuals come from talented families, suggesting that genetics is partially responsible for the physical, physiological or anthropometric characteristics that are needed for attaining athletic success [2]. Indeed, elite athlete status is partially heritable, with twin studies suggesting that 30–80% of the variance in this trait is explained by heritable factors [3]. Recent advances in genetic technology have allowed for greater exploration of the genetic underpinnings of elite performance, leading to the identification of single nucleotide polymorphisms (SNPs) and other genetic variants with the potential to influence sports performance, either directly or indirectly. For example, a SNP in *ACTN3*, R577X (rs1815739), has been shown to modify the attainment of elite speed–power athlete status [4,5]. Here, a common C-to-T base substitution results in the transformation of an arginine base (R) to a premature stop codon (X). X allele homozygotes are deficient in the protein encoded for by *ACTN3*, α-actinin-3, which is expressed exclusively in fast twitch muscle fibres. As a result, XX genotypes tend to have lower proportions of fast-twitch muscle fibres [6] and, because fast-twitch muscle fibres are an important component of speed–power performance, these genotypes might be underrepresented in elite speed–power cohorts [7]. The first study on this topic was conducted by Yang and colleagues [7], who reported that the X allele was significantly less frequent in elite male and female sprint athletes than in non-athletic controls and elite endurance athletes. Whilst in Caucasian populations the frequency of the XX genotype is ~20%, in Yang and colleague’s [7] cohort of Caucasian power Olympians, the XX genotype was entirely absent. The finding of significantly lower frequencies of the X allele and XX genotypes in elite speed–power athletes has been replicated [8,9], although equivocal findings have also been reported elsewhere [10]. Consequently, despite generally being estimated to explain around 1–3% of the variance in speed–power phenotype [2,11,12], *ACTN3* has subsequently been labelled a “gene for speed” [13,14,15]. *ACTN3*, however, is not the only gene associated with elite athlete status, with a recent review reporting that at least 155 genetic markers are potentially linked to elite athlete status, at least in some cohorts or ethnicities [16]. 

Whilst many of the currently established SNPs associated with elite athlete status are associated with physiological traits such as aerobic capacity, muscle strength or speed, there is the potential that other SNPs may exert a less direct—but no less important—effect on the attainment of elite sports performance. For example, both height [17] and body mass index are highly heritable [18], and both likely contribute to the achievement of elite athlete status on a sport-by-sport basis. Furthermore, psychological traits are also genetically influenced, with a number of SNPs associated with anxiety [19]. Interestingly, it has been found that variants in genes coding for proteins potentially modulating activity of brain emotion centers, particularly in genes encoding elements of serotoninergic, catecholaminergic and hypothalamic–pituitary–adrenal systems might also influence the predisposition to elite sports performance [20]. As such, the genetic influence on sporting performance is broad, multi-factorial, and pervasive. 

**Figure 1:**
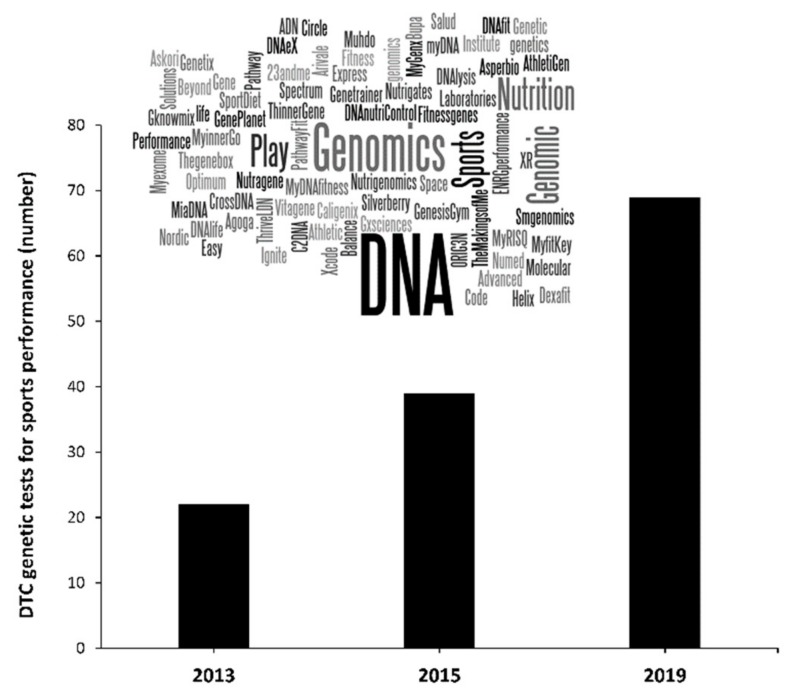
Number of companies that offer direct-to-consumer (DTC) genetic testing marketed as being related to sport performance, exercise performance and sports injury risk. Data in 2019 has been obtained by using the search terms “genetic”, “test”, “exercise” and “sport” in two popular search engines (Google and Bing) replicating the procedures followed by Williams et al., in 2013 [23] and Webborn et al., in 2015 [21].

Given the wide-ranging and potential influence of genetic variations on the attainment of elite athlete status, there is considerable interest in collecting genetic information to identify athletes with the potential to achieve elite status. Indeed, the number of companies that offer direct-to-consumer (DTC) genetic testing to predict sports performance/injury risk associated with sport has rocketed in recent years (Figure 1). Moreover, contemporary reports detail the use of genetic testing within the talent identification process in a number of countries and for a wide-range of sport enthusiasts [21]. One of the concerns with the use of such DTC genetic testing is that the information provided by the companies is based on a very limited genetic analysis, while the misuse of the research evidence to endorse the utility of this type of testing for early talent selection is common. The pace of growth in the number of companies that offer DTC genetic testing occurs partially due to the absence of worldwide agreements for a common regulation on this type of genetic testing. For these reasons, the general consensus amongst researchers is that these tests should not be used for talent identification [21], and the use of such tests in this way is ethically troubling [22]. Here, we discuss why current genetic tests cannot predict future sporting success, and explore the necessary advancements required to enable the utilisation of genetic information to accurately identify future talented performers.

## 2. Why We Cannot Currently Use Genetic Information for Talent ID

As previously mentioned, over 155 genetic markers have been tentatively linked with elite athlete status [16], although the association of most of these genetic variants to sports performance has a weak scientific background. The various suggestive genetic variants are typically—but not always—divergent, such that they predispose towards an increased chance of success in either power–strength or endurance sports/events, but not both. These divergent effects demonstrate that there is not a singular genetic profile that confers sporting success, but that the required genetic profiles are likely specific for characteristics of each sport. Whilst some of these markers, such as the aforementioned *ACTN3* rs1815739 [5,7], and to a lesser extent angiotensin-converting enzyme (*ACE*) insertion (I)/deletion (D) polymorphism (rs1799752) [5,24], and peroxisome proliferator-activated receptor gamma coactivator 1-alpha (*PPARGC1A* or simply *PGC1-1α*) rs8192678 [25,26] have been replicated in some studies; others, such as transcription factor A, mitochondrial (*TFAM*) rs1937, have yet to be satisfactorily replicated [27]. Of the 155 genetic variants identified as linked to elite athlete status [16], 31 have been associated with elite athlete status in at least two studies, with 12 replicated in at least three [16]. Accordingly, at present, we know only a few of the genetic markers that likely associate with elite athlete status, making predictions of future sporting prowess based on such information not only difficult but also likely inaccurate. In addition, testing the utility and efficacy of genetic testing in the talent identification process should be conducted to ascertain whether the information provided by genetic testing—and not obtained through other traditional non-genetic tests such as physical testing—is of relevance to increase the specificity of overall talent selection [28]. 

Another issue is that the currently available markers appear to offer poor specificity and sensitivity as talent identification tools. Returning to *ACTN3* rs1815739, whilst the R allele is associated with elite athlete status in speed–power sports events [5], with the XX genotype potentially playing a negative role in performance in such types of events [7], it remains unclear how discriminatory this information might be. In Caucasians, for example, ~80% of individuals possess an R allele [7]. In some black African populations, this percentage can be as high as 99% [29]. In a study of elite US and Jamaican sprinters—the populations providing the fastest eight 100 m runners of all time—there was no significant difference in *ACTN3* genotype frequencies between these athletes and their non-athletic peers, with 97% of non-athletes possessing at least one R allele [10]. Therefore, whilst the R allele may be required for elite sprint performance, as the vast majority of the world’s population possess it, knowledge of an individual’s *ACTN3* genotype is not particularly useful from a talent identification perspective. Furthermore, there are exceptions to the belief that an R allele is required for elite speed–power performance. In their study of elite sprinters, Papadimitriou and colleagues [12] reported that one male and one female 100 m sprinter, both of whom achieved the Olympic qualifying standard, did not possess an R allele. Additionally, Lucia and colleagues [30] reported the case of a long-jumper with a personal best of 8.26 cm, just 5 cm off the gold medal winning jump at the 2012 Olympic Games, who had the XX genotype. Such findings demonstrate that the lack of an *ACTN3* R allele does not preclude attainment of elite status in speed–power events. Additionally, performance-enhancing polymorphisms may still have a low prevalence in elite athlete populations. For example, a SNP (rs7181866) in nuclear respiratory factor 2 (*NRF2*) gene has been associated with elite athlete status, with a significantly higher proportion of the AG genotype compared to the AA genotype found in elite endurance athletes when compared to controls [9]. However, only 12–14% of these elite athletes possessed the “ideal” AG genotype, illustrating that the vast majority of elite athletes did not have this specific performance-enhancing polymorphism, again potentially limiting its use as a discriminating screen.

Such findings demonstrate the difficulties of a single gene approach for talent identification, highlighting that such an approach is no longer viable. In light of these findings, researchers have turned to Total Genotype Scores (TGS), whereby a number of elite athlete-associated SNPs are combined into a single polygenic score. Ruiz and colleagues [31,32] utilised this approach involving elite endurance and power athletes. For their study focusing on endurance athletes, they combined seven polymorphisms into a total score, finding that the mean score was higher in the athlete group than in the control (non-athletic) group [31]. This finding was replicated using a TGS comprised of six SNPs for power athlete status, with elite power athletes having a higher score than both endurance athletes and non-athletic controls [32]. Possession of the “perfect” polygenic profile (i.e., the elite athlete genotype of all SNPs) was rare, occurring in only 9.4% of the power athletes, and in no endurance athletes. In addition, there was considerable overlap between groups, such that a number of controls had better TGS than some elite power and endurance athletes [32]. Furthermore, whilst a TGS may help in discriminating between athlete and non-athlete, Santiago and colleagues [33] demonstrated that, in a group of rowers, it did not distinguish between different levels of performance (i.e., World vs National medallists). Earlier work [34,35] demonstrates that there is considerable similarity in polygenic scores within humans—athletes and non-athletes alike—when a low number of markers (in this case, 22 and 23) are used, such that, again, this approach would likely have limited real world specificity and sensitivity. Lastly, the configuration of TGS to differentiate between populations of athletes usually confers the same score to each SNP while the individual influence of each gene to the status of elite athlete is likely uneven, with some genetic variants exerting a greater influence than others. In order to improve the insights provided, a far greater number of performance-enhancing polymorphisms are likely required, whilst the relative weighing of the influence of each SNP may add further value in the development of increasingly accurate TGS. As such, further work should seek to identify additional SNPs associated with elite athlete status across a variety of sporting phenotypes, and replicate existing SNPs such that there is a pool of SNPs associated with elite athlete status that researchers have confidence in. The relative contribution of each SNP towards elite athlete status could then be determined via assessing the predictive ability of differently weighted algorithms, which can then be tested in hold-out cohorts for validation. 

As a summary of the above discussion, it seems clear that the provision of elite athlete status is a highly complex, polygenic trait, and that we know very few of the genetic variations that contribute to such a trait. As a result, it appears a fundamental requirement that, if genetic testing is to be utilised for talent identification purposes, a far greater number of polymorphisms associated with elite athlete status needs to be uncovered and then combined into a complex TGS model. 

## 3. What Further Knowledge Do We Require to Potentially Use Genetic Information for Talent Identification?

### 3.1. Genome-Wide Association Studies

The evolution of Genome-Wide Association Study (GWAS) methodology potentially offers an opportunity to expand the number of genetic variants currently associated with elite athlete status, or to other traits associated with sporting success, such as the likelihood of suffering performance-limiting injuries [36]. Whilst the majority of the SNPs currently associated with elite athlete status were elucidated via gene-association studies or candidate gene analysis—where a limited number of SNPs are hypothesised to have an effect on some particular trait associated with exercise performance, and that hypothesis is tested—GWAS are hypothesis-free. In a GWAS, a large number of SNPs are analysed for association with a specific trait. Given that there is no hypothesis to be tested, it provides a robust method to detect novel associations. However, due to the very low p-values required to reach genome-wide significance (*p* < 5 × 10^−8^), and the (often) very low effect sizes of any SNP, GWAS studies require very large subject numbers and might fail to identify some variations with a small effect. This is problematic when it comes to research on elite athletes, who are, by definition, rare. This problem was encountered in a GWAS carried out in multiple cohorts of elite endurance athletes by Rankinen and colleagues [37]. The authors utilised a cohort of elite Caucasian endurance runners (*n* = 315) and controls, along with a cohort of elite Japanese runners (*n* = 60) and controls, for the discovery phase of the GWAS. Following this discovery phase, in which no SNP met genome-wide significance, forty-two suggestive SNPs were taken through to a replication phase involving endurance athletes and controls from seven other countries, and from different ethnicities. Again, no genome-wide significant SNPs were found, although upon meta-analyses, a single SNP, rs558129 in polypeptide N-Acetylgalactosaminyltransferase 6 (*GALNTL6*) gene, was significant, with the T allele less frequent in athletes than in controls. As such, the authors summarised that there appeared to be no common SNP associated with elite endurance athlete status across this cohort, although they acknowledged their low sample size as a major limiting factor. This limitation is difficult to overcome and represents a significant roadblock in the search for genetic variants associated with elite performance. One potential method is to conduct a GWAS-approach analysis in a cohort of interest, and then attempt to replicate the suggestive findings (i.e., those of suggestive, but not genome-wide, statistical significance) of this analysis in additional, hold-out cohorts. Whilst novel, this approach has only been used in few sport genetics studies [38,39], despite its potential promise. A further potential roadblock is that there may be different associations between SNPs and elite performance across ethnicities, such that a SNP may be performance enhancing in Caucasians, but not in East Asians, for example, thereby requiring the development of ethnicity-specific SNP panels for the purpose of talent identification.

### 3.2. Rare Variants

A further avenue for exploration is that of rare gene variants that may predispose to elite performance. Generally, research focuses on common variants present in >1% of the population (i.e., polymorphisms) [2]. However, there are a few identified genetic variants which are very rare, and yet have the potential to positively affect performance. One such variant occurs at rs121917830 within the erythropoietin receptor (*EPOR*) gene. This variation is linked to a disease called erythrocystosis-1, where affected people have increased red blood cell production, and hence greater oxygen carrying capacity [2]. This outcome is potentially advantageous for endurance sports, and in fact one elite athlete, the Finnish cross-country skiing champion Eero Mantyranta, is known to possess this variant [40]. Preliminary data suggest an additional example of a rare variant, this time affecting the lamin/AC (L*MNA*) gene—related to muscular dystrophy—that was found in Canadian sprint hurdler Priscilla Lopes-Schliep [41]. Finally, a rare variation in the myostatin gene (*MSTN*) has been reported, in which the carrier was described as “extraordinarily muscular” [42]. Such a variation would perhaps be advantageous in sports/events demanding high levels of strength and muscle mass. One issue with the exploration of rare performance-enhancing variants is that, given their very low frequency, they can be hard to identify. In addition, studies of rare variants would need very large samples of unrelated individuals to correctly identify the influence of the variant on athletic performance. Lastly, some variants might also predispose to disease states; a factor raising complex moral and ethical questions. However, despite these potential issues, research continues towards their identification [41], not least because identification of individuals with disease-causing variants but presenting with a good health status could provide information relating to the underpinning mechanisms of these diseases and provide insight into remedial and resilience-building strategies [43]. 

### 3.3. A Predictive Threshold

An area of potential previous confusion is the number of performance-enhancing polymorphisms an individual may require before they are capable, from a genetic standpoint, of elite performance. Both Williams and Folland [34] and Hughes and colleagues [35] report that, in panels containing 23 (endurance) and 22 (strength–power) polymorphisms, the chances of one athlete possessing all was vanishingly small. As such, it seems unlikely that a single athlete possesses the “perfect” genetic profile, and it could be argued that having such a perfect profile is arguably unnecessary. Instead, athletes will likely possess a given number of performance-enhancing polymorphisms. Crucially, the polymorphisms possessed will differ between athletes, such that there might be only minimal overlap between individuals. In this way, once a large number of SNPs responsible for driving elite athlete status are uncovered—should such discovery ever occur—there will be the potential for the development of a threshold number, whereby possession of polymorphisms above this number would be associated with elite performance. Accordingly, there will not necessarily be a commonality in terms of the genetic variants present, although some crossover will certainly occur; instead, the main driving factor will be the total frequency of performance-driving variants. These SNPs will also likely differ between ethnicities, and so ethnicity-specific thresholds and genetic panels will be required. The utilisation of a large number of SNPs reduces the reliance on individual SNPs that occur either at high frequencies across populations (such as the *ACTN3* R allele), or those that, whilst linked to elite athlete status, are still present at relatively low frequencies in elite cohorts (for example the *NRF2* G allele). 

### 3.4. Epigenetic Modifications

**Figure 2:**
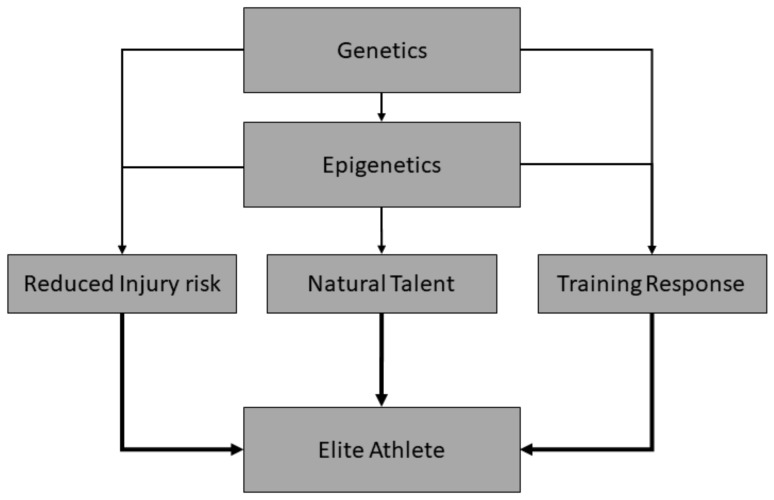
Influence of genetics and epigenetics on traits associated with elite performance. To succeed in sport, an athlete must possess genetic and epigenetic variations that might predispose to a natural talent trait (direct effect), and/or to enhanced response to physical training, and/or to reduced risk of injury (indirect effect).

In addition to genetic variations that might predispose, at least partially, to elite performance in a given sports, favourable epigenetic modifications that predispose to a natural talent trait (direct effect), and/or enhanced response to physical training and reduced risk of injury (indirect effect; (Figure 2)) would also likely be advantageous. In this context, epigenetic modifications—changes in genetic expression that are not due to variations in the underlying genetic code—may modify the attainment of elite athlete status [44] and represent another source of heritable variation that might help in sports talent identification [2]. These epigenetic changes are typically comprised of DNA methylation, histone modifications, and non-coding RNAs, particularly microRNAs (miRNAs) [44,45]. For example, miRNAs are involved in post-transcriptional control of messenger RNA (mRNA) and they include a highly complex network that regulates protein expression within the cell. Sequence and functional conservation of many human miRNAs with distantly related organisms suggests their crucial role in cellular progress [46]. Given their fundamental role in cell functioning, miRNAs have been shown to influence both skeletal muscle differentiation [47] and the magnitude of exercise-induced adaptations [48], which are important components of the journey towards elite athlete status. The use of epigenetic information might help to discover individuals with high chances of possessing characteristics associated with elite sport performance, but also to understand what and how epigenetic modifications can be modulated through appropriate training and dietary regimes to improve performance [2]. As of yet, whilst it appears that epigenetic changes may modify the response to exercise [44,45], it is not clear what overall effect they may have on the attainment of elite status. Additionally, such modifications have the potential to be passed down through generations [49], and thus might form part of the heritable aspect of elite athlete status, although the evidence that this actually occurs in humans is currently weak. Nevertheless, the ability to test for these changes, which are often tissue-specific, could assist in the identification of talented athletes; however, the quantification of epigenetic modifications can, in some cases, be challenging. Many epigenetic modifications are often both tissue specific and transient [50]; as a result, quantification would require frequent tissue sampling, which in the case of muscle would be invasive and traumatic, and in the case of other tissue, such as the heart and brain, highly impractical [51]. However, early research suggests that salivary profiling of DNA methylation markers holds promise [52], making this approach potentially more attractive and practically realistic. 

### 3.5. Lessons from Disease Prediction

One area where the use of genetics to make informed predictions of a future event has been well explored is that of disease risk. Whilst some diseases, such as cystic fibrosis, are monogenic, most diseases are complex and polygenic in nature [53]. Similar to elite athlete status, whilst many diseases have been shown to have a large genetic component, the disease-causing variants identified to date explain little of the variance between individuals; an issue referred to as the ‘missing heritability’ problem [54]. One suggested approach to overcome the problem of missing heritability is to lower the threshold for discovery of SNPs affecting the trait of interest. Due to the high number of comparisons carried out in a GWAS, statistical significance for discovery of new variants is set at *p* < 5 × 10^−8^. However, the lowering of this threshold has been shown to lead to explanation of a greater proportion of heritability [55]. Recently, Khera and colleagues [56] utilised a TGS comprised of 6,630,150 polymorphisms to create a risk score for coronary artery disease that had an area under the curve of 0.81, suggesting a strong predictive ability; many of these polymorphisms had miniscule effect sizes and weak significance, and yet combined to produce a powerful predictive tool. Such a method clearly holds promise for traits that have a large—but poorly elucidated—genetic component [57], such as elite athlete status. Indeed, returning to the recent GWAS on elite endurance status, whilst no SNP was discovered at the genome-wide significance level (*p* < 5 × 10^−8^), a number of SNPs had suggestive significance, and so may hold predictive ability as part of a TGS. As such, it appears that, in order to successfully predict future elite athlete status, models involving multiple genetic variants with low effect sizes are likely required, although this approach has recently been criticised from a disease-prediction viewpoint [58]. However, we return to the common issue of sample size; for discovery of relatively common genetic variants with small effect sizes, sample sizes in excess of 10,000 subjects are likely required [59]—a number considerably greater than that of all truly elite athletes within a specific performance trait on the planet. 

## 4. Even If We Could Test for Talent—Should We?

There are serious and well-placed concerns about the use of genetic information, either alone or in combination with existing measures, for talent identification within sport [21]. It is generally considered that, within sporting contexts, genetic testing should not be carried out on athletes younger than 18 years of age [21]. If genetic testing for talent does become evidence-based, then there will be a requirement for the development of guidelines on its use, in part to protect vulnerable young children. For example, should sports clubs be able to demand players undergo genetic testing as part of their talent identification programmes? Who should have access to the genetic data? Will it be used in a discriminatory way? What if a player refuses to undergo a genetic test? What happens if testing uncovers a potential disease-causing variant? The latter point is potentially an important issue, as whilst it could lead to health-promoting medical interventions, it could also lead to unnecessary medical explorations, as well as increased worry, both on the part of the athlete tested and their relatives, who may also carry the disease-causing variant. Such outcomes are highly unpalatable, and likely represent an extreme example, but demonstrate the potential misuse of genetic information for talent identification. 

If and when genetic testing is used to predict future elite athlete status, there will be many false positives and false negatives (i.e., many individuals will be misattributed to future elite or non-elite status [60]). Whilst such error rates may be acceptable at a population level, they are obviously ethically troubling at an individual one—who has the right to tell a young athlete that they do not have the genetic potential to succeed in a given sport? Perhaps more importantly, what impact would receiving this information have on that individual’s future exercise behaviour, which, given the wide-ranging health benefits of exercise [61], is an important consideration for lifelong health. For these reasons, some authors have suggested that emphasis in the use of genetic testing for talent identification in children needs to be focused on the wellbeing on the child in the present, not on the potential of future benefits that may never arrive [22]. 

## 5. What Could We Potentially Use Genetic Testing for?

It has previously been argued that, instead of using genetic information for talent identification in the traditional sense, this information could be used to match individuals to the type of training to which they are most suited, and from which they will elicit the greatest adaptations [62,63,64]. Additionally, genetic information could be utilised to identify those athletes with an increased risk of injury, allowing for the provision of pre-emptive strategies to reduce that risk. For example, Varley and colleagues [65] identified a number of polymorphisms associated with an increased stress fracture risk in a cohort of elite athletes; in this case, the high-risk athletes could undergo additional bone mineral density monitoring, along with targeted interventions, such as vitamin D and calcium supplementation. 

Whilst traditional research has focused on the physiological drivers of elite athlete status (reviewed in detail by Ahmetov and colleagues [16]), there is the potential to explore the genetic underpinnings of psychological factors, such as anxiety, stress resilience, and skill acquisition. This has not yet been explored in detail, although a SNP in catechol-O-methyltransferase (*COMT*) gene, rs4680, has been linked with competition performance in swimmers [66] and with personality traits in ultra-endurance athletes [67]. Catechol-o-methyltransferase plays a role in the regulation of dopamine within the prefrontal cortex [19]; variation in this SNP affects dopamine levels and thus information processing and memory [19]. Emerging research has also shown an association between a number of polymorphisms and the skill acquisition process [68]. Finally, a number of genetic variants have been linked to an increased susceptibility to concussion injuries [69]. As a result, whilst this information could be used to bias against those with the perceived “unfavourable” genotypes, it could also be used to personalize the training process, identifying those athletes who need greater attention in these areas. Furthermore, with regard to both injury and concussion, the information could be used to better inform preventative methods, along with increasing the personalisation of recovery and return-to-play protocols, particularly given the evidence that genetic information might enhance adherence [70]. That said, similar to the use of genetic information as a talent identification tool, the research in this area is currently under-developed, and plagued with similar issues to those outlined in this manuscript. The early and initial studies exploring the use of genetic information to training programme design and management, whilst promising, require replication in different cohorts and larger samples to confirm their findings. 

## 6. Conclusions

Although there is a well-established effect of genetics on the attainment of elite athlete status, based on the available evidence, it is clear that the current use of genetic tests for the prediction of future elite athlete status is ineffectual, a finding that echoes recent consensus statements [21,71]. Despite the increasing availability of commercial DTC genetic tests, the currently available data suggest the use of the information provided by these tests for talent identification or selection, especially in children, is unfounded. In order to be able to use genetic information within the talent identification process, a far greater number of performance-enhancing polymorphisms needs to be both discovered and replicated in subsequent studies. Future studies in elite athletes must contain detailed information on phenotypes associated with sporting performance, in conjunction with obtaining samples such as blood or muscle tissue that help identify the association between genetics and epigenetics. The combination of performance-enhancing polymorphisms into a TGS, especially if the evidence threshold is lowered, appears to offer a solution to the currently limited predictive capabilities of small numbers of genetic variants. As the evidence base grows, we believe it will be possible to determine a TGS threshold, above which an individual’s chance of achieving elite athlete status in a given sport or event is higher. However, we are also of the opinion that there will always be individuals with a score below this threshold who go on to achieve elite athlete status, and those with scores above the threshold who will not be elite athletes. Because elite athlete status is a manifestation of a number of variables, not just genotype, it seems unlikely that it will ever be possible to use genetic information to unequivocally identify a future elite athlete. At best, genetic information may represent a potentially useful adjunct to existing talent identification procedures, enhancing the process of selection, particularly as genetic information is not subject to some of the issues that commonly plague traditional talent identification processes, such as maturation and the relative age effect. Additionally, it has previously been argued that genetic information may be used in the future to identify those with the greatest potential to show favourable adaptations to training [62,64], as well as determine the optimal training type to elicit such adaptations [63]. Furthermore, there is the potential to utilise such information to reduce injury occurrence [72]. Again, and we must be clear; such information should not be used as a standalone, but as an adjunct to current talent identification processes, thereby allowing the training process to become more personalised, and enabling athletes to get ever closer to their maximum potential.
